# Upregulation of YPEL3 expression and induction of human breast cancer cell death by microRNAs

**DOI:** 10.1007/s43188-024-00251-2

**Published:** 2024-06-21

**Authors:** Boyoung Lee, Yeo-Jung Kwon, Sangyun Shin, Tae-Uk Kwon, Hyemin Park, Hyein Lee, Ji-Heung Kwak, Young-Jin Chun

**Affiliations:** College of Pharmacy and Center for Metareceptome Research, Chung-Ang University, 84 Heukseok-Ro, Dongjak-Gu, Seoul, 06974 Republic of Korea

**Keywords:** miR-34a, miR-605-5p, MDM2/4, YPEL3, YAP1

## Abstract

MicroRNAs (miRNAs), molecules comprising 18–22 nucleotides, regulate expression of genes post-transcriptionally at the 3′ untranslated region of target mRNAs. However, the biological roles and mechanisms of action of miRNAs in breast cancer remain unelucidated. Thus, in this study, we aimed to investigate the functions and possible mechanisms of action of miRNAs in breast cancer to suppress carcinogenesis. Using miRNA databases, we selected miR-34a and miR-605-5p to downregulate *MDM4* and *MDM2*, respectively, because these ubiquitin E3 ligases degrade p53 and promote carcinogenesis. Results showed that miR-34a and miR-605-5p suppressed MDM4 and MDM2 expression, respectively. Moreover, they reduced the expression of yes‑associated protein 1 (YAP1), a well-known oncogene involved in Hippo signaling, but upregulated the mRNA and protein expression of yippee-like 3 (YPEL3). To elucidate whether these miRNAs promote cellular senescence and death through YPEL3 upregulation, we examined their effects on cellular proliferation, SA-β-gal activity, and mitochondrial activity in human breast cancer MCF-7 cells. Given their upregulating effect on YPEL3 expression, miR-34a and miR-605-5p increased the number of β-galactosidase-positive cells and depolarized live cells (by 10%–12%). These data suggest that miR-34a and miR-605-5p promote cellular senescence and cell death. Thus, they may act as tumor suppressors by inducing Hippo signaling and may serve as novel therapeutic agents in breast cancer treatment.

**Supplementary Information:**

The online version contains supplementary material available at 10.1007/s43188-024-00251-2.

## Introduction

P53 is a tumor suppressor protein that functions in various processes, including DNA repair, cell cycle regulation, cell aging, and apoptosis, and plays a key role in tumor prevention. In the normal state or absence of stress in the cell, MDM2 degrades and inhibits p53. P53 is activated in response to cellular stresses, such as DNA damage, aberrant cell cycle, hypoxia, and oxidative stress [1]. Sustained p53 activation prevents and repairs DNA damage. P53 initiates various cellular responses by selectively regulating target genes in response to stress. Thus, p53 must be tightly regulated by other factors to avoid unnecessary activation. MDM2 and MDM4 are E3 ubiquitin ligases that suppress p53 activation, making them essential negative regulators of p53 [2].

MDM2 binds to and degrades p53 through ubiquitination. Therefore, MDM2 is important for balancing p53-mediated tumor suppression. Disruption of this balance may impair the function of p53 and promote tumorigenesis. MDM2 overexpression is another molecular mechanism that inactivates p53 during cellular tumorigenesis [3].

MDM4 is an MDM2 homolog that inhibits p53 in vivo, suggesting its important role in cancer development [4]. Recent biochemical studies have shown that the MDM4 RING domain plays an essential role in the polyubiquitination and degradation of MDM2-dependent p53 [5]. MDM2 and MDM4 bind to each other and play nonoverlapping roles in regulating p53 activity. MDM4 expression is increased in patients with hepatoblastoma, and this phenomenon is correlated with decreased expression of the p53 target gene [6]. Although MDM2 alone can inhibit p53, heterodimerization of MDM2 and MDM4 plays a critical role in p53 inhibition [7]. This complex acts more effectively than MDM2 alone, and MDM4 accelerates the MDM2-mediated degradation of p53 [8]. High expression of MDM4 and MDM2 occurs frequently in human cancers, including breast cancer, gliomas, soft tissue sarcomas, and melanomas [9]. These proteins are overexpressed in various tumors and are considered oncogenic; thus, their expression must be properly regulated to prevent cancer development [10].

MicroRNAs (miRNAs), small non-coding RNAs that consist of 18–22 nucleotides and are endogenously expressed, play a major role in the expression of target genes [11]. These molecules are located in target mRNAs and frequently found in the 3ʹ untranslated regions (3ʹ-UTRs). Although the binding sites of miRNAs and target genes have been estimated from various bioinformatics data, the functions of all the estimated miRNA binding sites are not completely understood [12]. Endogenous miRNAs have attracted considerable research attention because they influence several biological processes in the human body and demonstrate strong biomarker potential. In patients with non-small cell lung cancer, the upregulation of miR-155 and downregulation of let-7 expression are associated with poor prognosis [13]. Members of the miR-29 family induce tumors in breast cancer and reverse aberrant methylation in lung cancer by targeting DNA methyltransferases 3A and 3B [14]. MiR-380-5p, which is highly expressed in mouse embryonic stem cells and neuroblastoma, is associated with poor prognosis of neuroblastoma patients [15]. Consequently, the relative levels of miRNAs affect mRNA expression, which plays an important role in carcinogenesis and other diseases [16].

Cellular senescence, in which cell division is restricted for a long period, is a key factor in human aging and can be induced in response to cellular stress and stimuli, such as DNA damage, cellular stress, telomere shortening, reactive oxygen species activation, and oncogene activation [17]. Cells under stress are usually damaged, which considerably increases the possibility of cancer development [18]. Cellular senescence can either be beneficial or detrimental depending on the biological function and type of the cell undergoing this phenomenon [19]. Moreover, it ultimately leads to apoptosis [20]. The accumulation of senescent cells decelerates tissue regeneration and causes inflammation, leading to aging and carcinogenesis. Despite these studies, the biological roles and mechanisms of action of miRNAs in breast cancer remain unclear to date.

Thus, in this study, we aimed to investigate the functions and possible mechanisms of action of miRNAs in breast cancer to suppress carcinogenesis. This study offers miRNAs that can serve as novel therapeutic agents in breast cancer treatment.

## Materials and methods

### Chemicals and reagents

Antibodies against MDM2 and MDM4 were purchased from Santa Cruz Biotechnology (Dallas, TX, USA). A polyclonal antibody for YPEL3 was obtained from Boster Bio (Pleasanton, CA, USA). RPMI 1640 medium and fetal bovine serum (FBS) were purchased from HyClone (Logan, UT, USA). Doxorubicin was obtained from Selleck Chemicals (Houston, TX, USA). The enhanced chemiluminescence (ECL) kit and bicinchoninic acid (BCA) protein assay kit were purchased from Thermo Fisher Scientific (Waltham, MA, USA). QGreenBlue 2X qPCR Master Mix was obtained from Cell Safe (Yongin, Korea), and D-Plus™ CCK cell viability assay kit was purchased from Dongin biotech (Seoul, Korea). All the chemicals and reagents used in the experiments were of the highest commercially available quality.

### Cell culture

Human breast cancer MCF-7 cells were purchased from Korea Cell Line Bank (Korea). The cells were cultured in RPMI 1640 medium supplemented with 10% (v/v) heat-inactivated FBS, 100 U/mL penicillin, and 100 μg/mL streptomycin at 37 °C in a humidified atmosphere of 5% CO_2_.

### Transient transfection of miRNA

MiRNA mimics were obtained from BIONEER (Daejeon, Korea), and the sequences were as follows: Hsa-mir-34a (UGUUGGUCGAUUCUGUGACGGGU), Hsa-mir-605-5p (UCCUCUUCCGUGGUACCCUAAAU). The cells were transfected with 80 nM miRNA mimics using the Neon™ transfection system (Invitrogen, Carlsbad, CA, USA) and cultured in antibiotic-free RPMI medium with 10% FBS for 48 h.

### RNA isolation, reverse transcription, and RT-PCR

After transfection, mRNAs and miRNAs were extracted using the Hybrid-${\text{R}}^{\text{TM}}$ miRNA kit (GeneALL, Seoul, Korea). Total RNA (1000 ng) was transcribed at 37℃ for 1 h in a 25-μl reaction volume containing RNase buffer, 10 mM dNTPs, RNase inhibitor, M-MLV reverse transcriptase, and 100 pmol of oligo-dT primers. The cDNA products were amplified using the Rotor-Gene SYB ${\text{R}}^{\circledR }$ PCR kit (Qiagen, Hilden, Germany). Each reaction contained 10 μl of 2 × SYB ${\text{R}}^{\circledR }$ Green PCR Master Mix, 2 μl of each oligonucleotide primer, and 2 μl of cDNA in a final volume of 20 μl. The amplification conditions were as follows: one cycle of 95 °C for 2 min, followed by 35 cycles of denaturation at 95 °C for 10 s, annealing at 58 °C for 15 s, and extension at 72 °C for 30 s. MiRNA (400 ng) was transcribed at 37 °C for 1 h in a 20-μl reaction volume containing 5 × miScript HiSpec Buffer, 10 × miScript Nucleics Mix, and miScript Reverse Transcriptase Mix (Qiagen, Seoul, Korea). The cDNA products were amplified using target-specific miScript Primer Assays and the miScript SYBR Green PCR kit with a final volume of 20 μl. The amplification conditions were as follows: one cycle of 95 °C for 15 min, followed by 40 cycles of denaturation at 94 °C for 15 s, annealing at each Tm 52–62 °C for 30 s, and extension at 70 °C for 30 s. The primer sequences used in this experiment are listed in Table 1.

**Table 1:** Primer sequences used in real-time qPCR

Gene	Forward primer (5ʹ-3ʹ)	Reverse primer (5ʹ-3ʹ)
*MDM2*	GAATCATCGGACTCAGGTACATC	TCTGTCTCACTAATTGCTCTCCT
*MDM4*	TGATTGTCGAAGAACCATTTCGG	TGCAGGGATCAAAAAGTTTGGAG
*YPEL3*	GTGCGGATTTCAAAGCCCAAG	CCCACGTTCACCACTGAGTT
*YAP1*	CGCTCTTCAACGCCGTCA	AGTACTGGCCTGTCGGGAGT
*Lamin B1*	GAAAAAGACAACTCTCGTCGCA	GTAAGCACTGATTTCCATGTCCA
*18S*	GTAACCCGTTGAACCCCATT	CCATCCAATCGGTAGTACG

### Western blotting

Cells were harvested and solubilized in ice-cold lysis buffer containing 50 mM Tris–HCl (pH 8.0), 0.1% sodium dodecyl sulfate (SDS), 1% Triton X-100, 0.5% sodium deoxycholate, 2 mM EDTA, 10 mM NaF, 150 mM NaCl, and 1 mM phenylmethylsulfonyl fluoride. The protein concentration was measured using BCA protein assay reagents. The extracted proteins (40 μg) were heated at 99 °C for 5 min, loaded onto 10%–15% SDS–polyacrylamide gel, and then electrochemically transferred onto polyvinylidene difluoride membranes. After being transferred, nonspecific binding to membranes was blocked with skimmed milk (5%) in Tris-buffered saline containing 0.1% Tween-20 (TBS-T) for 1 h at 4 °C. After overnight incubation with specific primary antibodies (1:1000 dilution), the membranes were washed thrice with TBS-T for 10 min each. Subsequently, the membranes were incubated overnight with secondary antibodies (1:5000 dilution) at 4 °C. Protein bands were visualized using the ECL method. The band intensity on the western blots was quantified and analyzed using ChemiDoc XRS (Bio-Rad, Hercules, CA, USA) and normalized to that of β-actin.

### Confocal microscopy

The cells after transfection of control or mimics for miR-34a and miR-605-5p were seeded on poly-D-lysine-coated coverslips. After incubation at 48 h, the cells were fixed with 4% (w/v) formaldehyde in PBS for 30 min at 25 °C. After washing with PBS, the cells were blocked for 40 min in PBS containing 5% goat serum and 0.2% Triton X-100. After overnight incubation with a 1:200 dilution of the primary antibodies, the cells were washed and stained with a 1:200 dilution of the secondary antibodies. After an additional wash process, the coverslips were mounted onto glass slides with 3 μL of UltraCruz™ Mounting Medium containing 4′,6-diamidino-2-phenylindole. Fluorescence signals were analyzed using an LSM800 Confocal Laser Scanning Microscope (Carl Zeiss, Jena, Germany).

### Cell viability assay

MCF-7 cells (5 × 10^3^ cells/well) were plated in 96-well microplates and incubated for 96 h at 37 °C. Before seeding, the cells were transfected with miR-34a or miR-605-5p mimics (80 nM). After incubation, the cells were treated with 10 μL of CCK solution (Dongin biotech) and incubated for 1 h at 37 °C. The produced formazan dyes were quantified by measuring the absorbance at 450 nm using a Tecan Sunrise™ microplate reader (Männedorf, Switzerland). All experiments were independently performed in triplicate.

### Senescence-associated β-galactosidase assay

MCF-7 cells (1 × 10^6^ cells/well) were seeded in 6-well plates after transfection with miRNA mimics. The cells were stained at 37 °C overnight with a staining solution in SA-β-galactosidase (gal) staining kit (Cell Signaling Technology, USA) following the manufacturer’s instructions.

### Analysis of MitoPotential function

Transfected cells were seeded at a density of 1 × 10^5^ cells. After harvesting with 0.1% trypsin–EDTA, the cell pellet was resuspended in the assay buffer of the MitoPotential kit (Millipore, Germany). The cells were stained with MitoPotential working solution for 20 min. After staining, the cells were incubated with 7-aminoactinomycin D for 5 min. MitoPotential function was measured using MUSE® Cell Analyzer (Millipore) following the manufacturer’s instructions.

### Bioinformatics analysis

A TNM plot analysis (https://tnmplot.com/analysis/) was performed to elucidate the expression of MDM4, MDM2, YAP1, and YPEL3 in breast cancer. Specific miRNAs targeting MDM4, MDM2, or YAP1 were found using the bioinformatic databases as candidates for further studies; miRTarBase (http://mirtarbase.cuhk.edu.cn/php/index.php), TargetScan (http://www.targetscan.org), and miRDB (http://www.mirdb.org).

### Statistical analysis

Statistical analyses were conducted using one-way analysis of variance, followed by the Dunnett’s multiple comparison and t tests, in Prism version 10.0.0 (GraphPad Software Inc., San Diego, CA, USA). Differences were considered statistically significant at *p* < 0.05.

## Results

### Expression of MDM4, MDM2, YAP1, and YPEL3 in breast *cancer* samples by tumor, node, metastasis (TNM) plot analysis

**Fig. 1:**
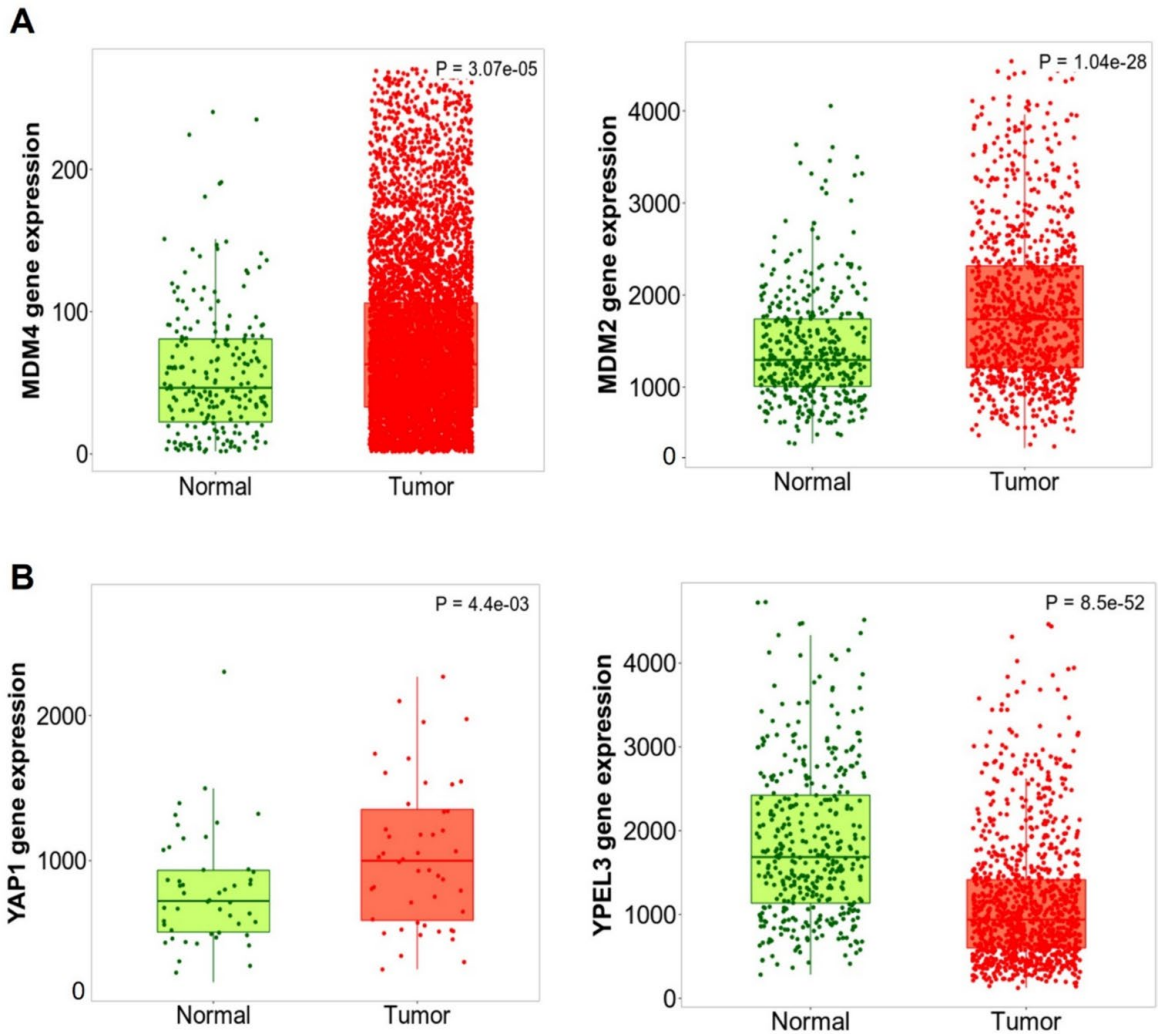
Expression of MDM4, MDM2, YAP1, and YPEL3 in human normal and breast tumor samples. **a** TNM plot data provided by Kaplan–Meier Plotter show the expression of MDM4 and MDM2 in normal and tumor samples in breast cancer. **b** Kaplan–Meier Plotter (TNM plot) shows the expression of YAP1 and YPEL3 in normal and tumor samples in breast cancer

The expression levels of MDM4, MDM2, YAP1, and YPEL3 in human breast tumor samples were investigated compared to normal samples using TNM plot analysis. The expression levels of MDM4 and MDM2 were higher in the tumor samples than in the normal samples (Fig. 1A). YAP1 expression was also high in the tumor samples, but YPEL3 levels were relatively lower in the tumor samples than in the normal samples (Fig. 1B).

### Selection of specific miRNAs to modulate MDM4 and MDM2 expression

**Fig. 2:**
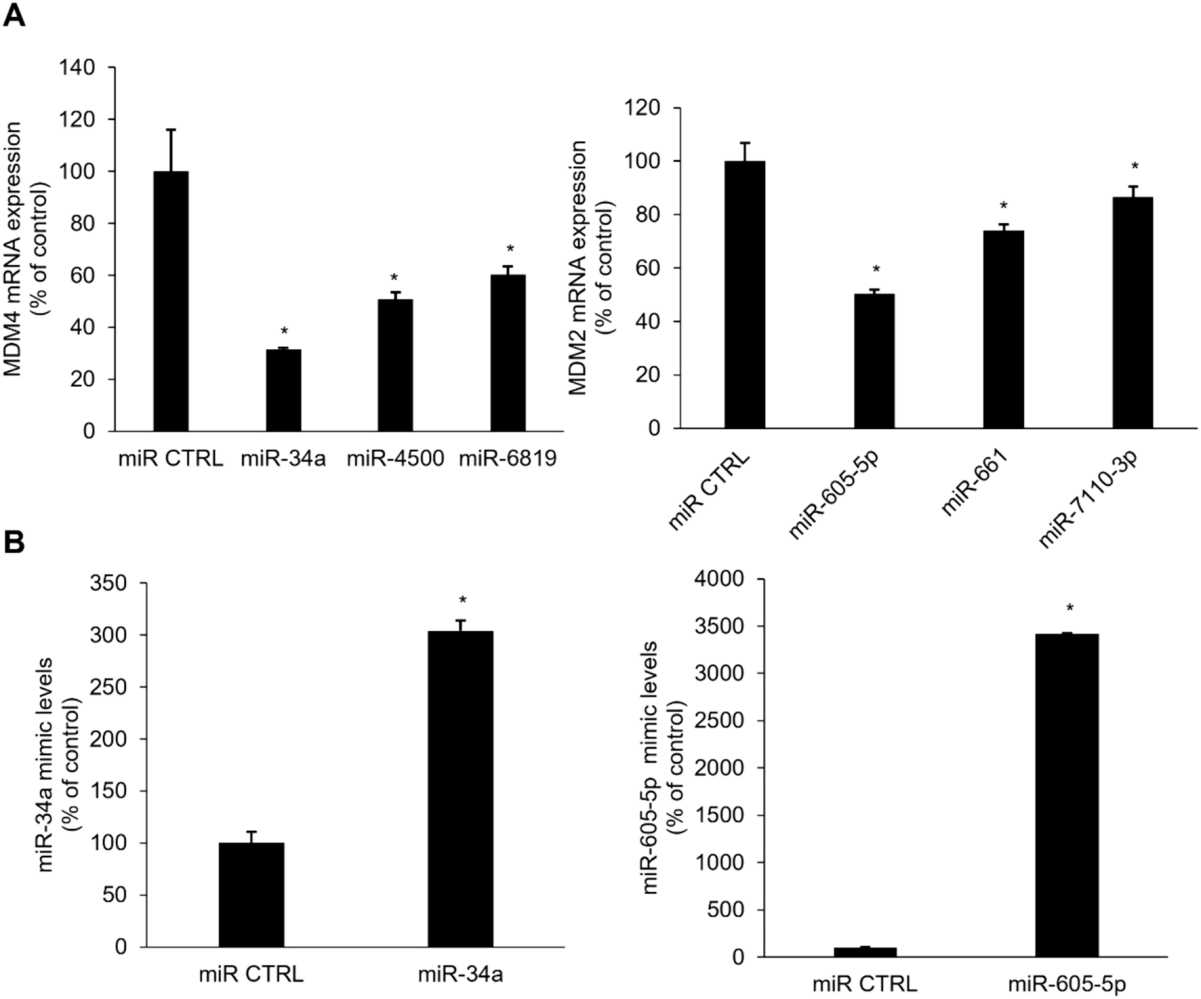
Suppression of MDM4 and MDM2 mRNA expression by miRNAs in MCF-7 cells. Real-time qPCR was performed to determine MDM4 and MDM2 mRNA expression or miRNAs expression using MCF-7 cells following transfection of mimics for miR-34a or miR-605-5p. Data represent the mean ± SD (*n* = 3) (*p < 0.05). **a** mRNA expression levels for MDM4 and MDM2, and **b** miRNA expression levels for miR-34a or miR-605-5p

**Fig. 3:**
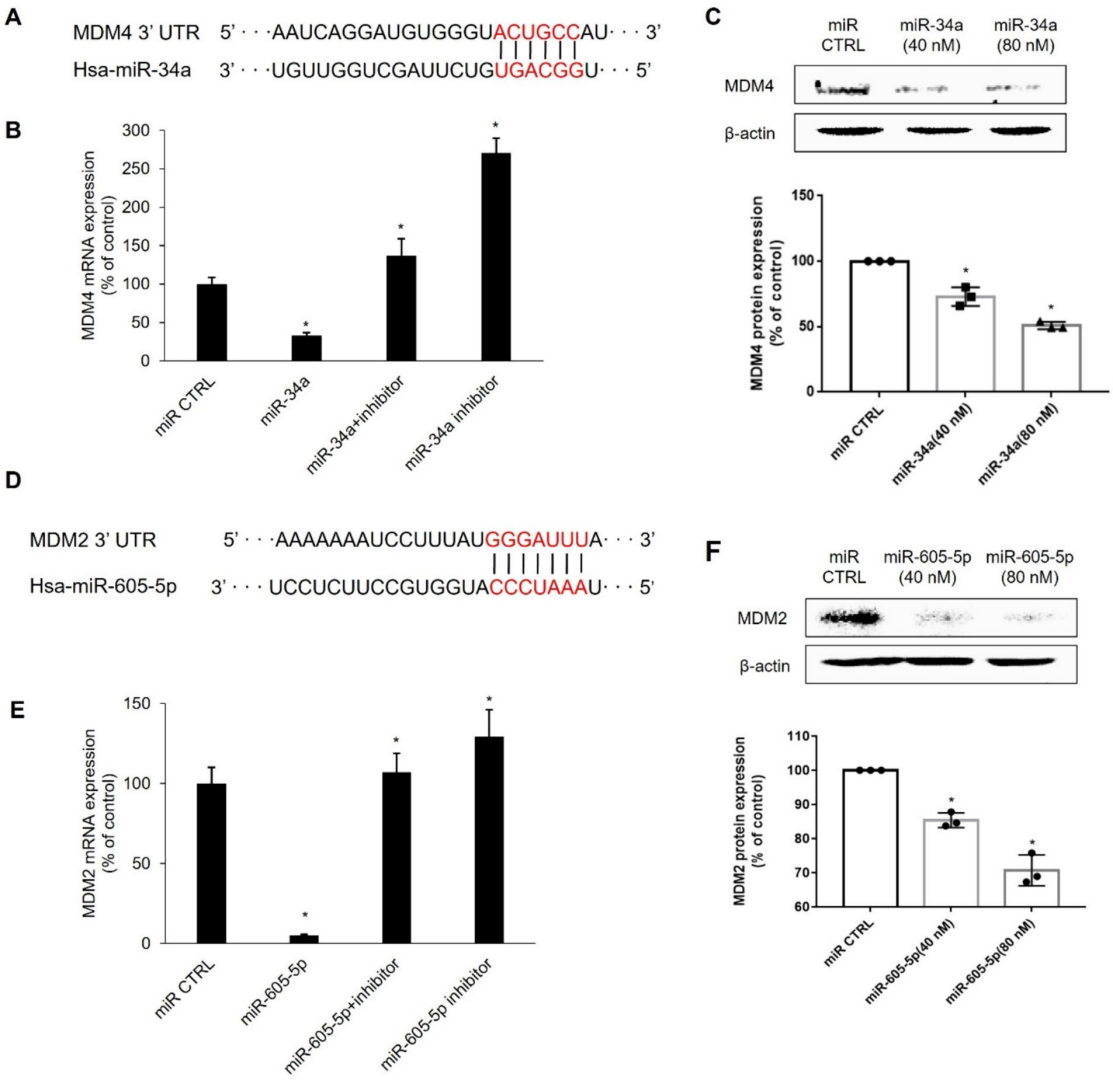
MiR-34a downregulates MDM4 mRNA and protein levels and miR-605-5p reduces MDM2 mRNA and protein levels. **a** Putative binding sites of miR-34a at the 3ʹ-UTR of MDM4 mRNA. Sequences of MDM4 and miR-34a bind complementarily in red-marked regions. **b** MiR-34a (80 nM) was transfected in MCF-7 cells in the presence or absence of miR-34a inhibitor. Data represent the mean ± SD (*n* = 3) (*p < 0.05). **c** MCF-7 cells were transfected with miR-34a (40 nM or 80 nM) and harvested for western blot. β-actin was used as loading control. Data represent the mean ± SD (*n* = 3) (*p < 0.05). **d** It indicates that the putative binding sites of miR-605-5p at the 3ʹ-UTR of MDM2. Sequences of MDM2 and miR-605-5p bind complementarily in red-marked regions. **e** MiR-605-5p (80 nM) was transfected in MCF-7 cells in the presence or absence of miR-605-5p inhibitor. Data represent the mean ± SD (*n* = 3) (*p < 0.05). **f** MCF-7 cells were transfected with miR-605-5p (40 nM or 80 nM) and harvested for western blot. β-actin was used as loading control. Data represent the mean ± SD (*n* = 3) (*p < 0.05)

MDM4 and MDM2 are crucial negative regulators of p53 expression. We performed a computational analysis using miRDB to investigate which miRNAs modulate MDM4 and MDM2 expression to affect p53 levels and their function in human breast cancer cells. Various miRNAs were screened based on their target scores to identify the miRNAs that significantly regulate the expression of MDM4 and MDM2. MiRNAs targeting MDM4, hsa-miR-34a, hsa-miR-4500, and hsa-miR-6819, the top three candidates with the highest scores both in miRDB and TargetScan, were selected. For MDM2, hsa-miR-605-5p, hsa-miR-661, and hsa-miR-7110-3p were selected (Table 2). To confirm the effects of the selected miRNAs, we transfected the cells with mimics of each miRNA. Of the three tested miRNAs, miR-34a significantly inhibited MDM4 expression. In addition, miR-605-5p significantly inhibited MDM2 expression (Fig. 2A). The levels of intracellular miRNAs were significantly elevated in the MCF-7 cells transfected with miR-34a or miR-605-5p (Fig. 2B). MicroRNA inhibitors, single-stranded RNA oligonucleotides complemented with mature miRNA sequences, prevent the corresponding miRNAs from binding to their target genes [21]. The binding sequences of hsa-miR-34a and MDM4 3ʹ-UTR were verified using the TargetScan database (Fig. 3A). MDM4 mRNA expression was significantly suppressed in the cells transfected with miR-34a (80 nM) for 48 h. Treatment with the miR-34a inhibitor recovered the miR-34a-mediated suppression of MDM4 expression. Importantly, treatment with the miR-34a inhibitor alone strongly increased MDM4 mRNA expression (Fig. 3B). Transfection with miR-34a downregulated MDM4 protein expression in a concentration-dependent manner (Fig. 3C). Similar to those of miR-34a, the binding sequences of hsa-miR-605-5p and MDM2 3ʹ-UTR were also verified (Fig. 3D). qRT-PCR was performed to confirm the miR-605-5p-mediated suppression of MDM2 expression. Treatment with the mir-605-5p inhibitor recovered the miR-605-5p-mediated suppression of MDM2 expression (Fig. 3E). Transfection with miR-605-5p (0, 40, and 80 nM) modulated MDM2 protein expression in a concentration-dependent manner (Fig. 3F). Overall, these results indicate that miR-34a and miR-605-5p are bona fide miRNAs targeting MDM4 and MDM2, respectively.

**Table 2:** Selection of microRNAs targeting MDM4 and MDM2 by miRNA target prediction databases

Gene	miRNA	Target score	
*MDM2*	hsa-miR-605-5p	miRDB	80
		TargetScan	92
		miRTarBase	Yes
	hsa-miR-661	miRDB	–
		TargetScan	81
		miRTarBase	Yes
	hsa-miR-7110-3p	miRDB	93
		TargetScan	82
		miRTarBase	Yes
*MDM4*	hsa-miR-34a	miRDB	100
		TargetScan	98
		miRTarBase	Yes
	hsa-miR-4500	miRDB	95
		TargetScan	98
		miRTarBase	Yes
	hsa-miR-6819	miRDB	85
		TargetScan	92
		miRTarBase	Yes

Candidate miRNAs which target MDM4 and MDM2 were predicted by miRDB, miRTarBase, and TargetScan. For MDM4, three high-scoring miRNAs candidates, miR-34a, miR-4500 and miR-6819 were selected respectively. For MDM2, miR-605-5p, miR-661, and miR-7110-3p were selected.

### MiR-34a and miR-605-5p positively regulate YPEL3 in MCF-7 cells

**Fig. 4:**
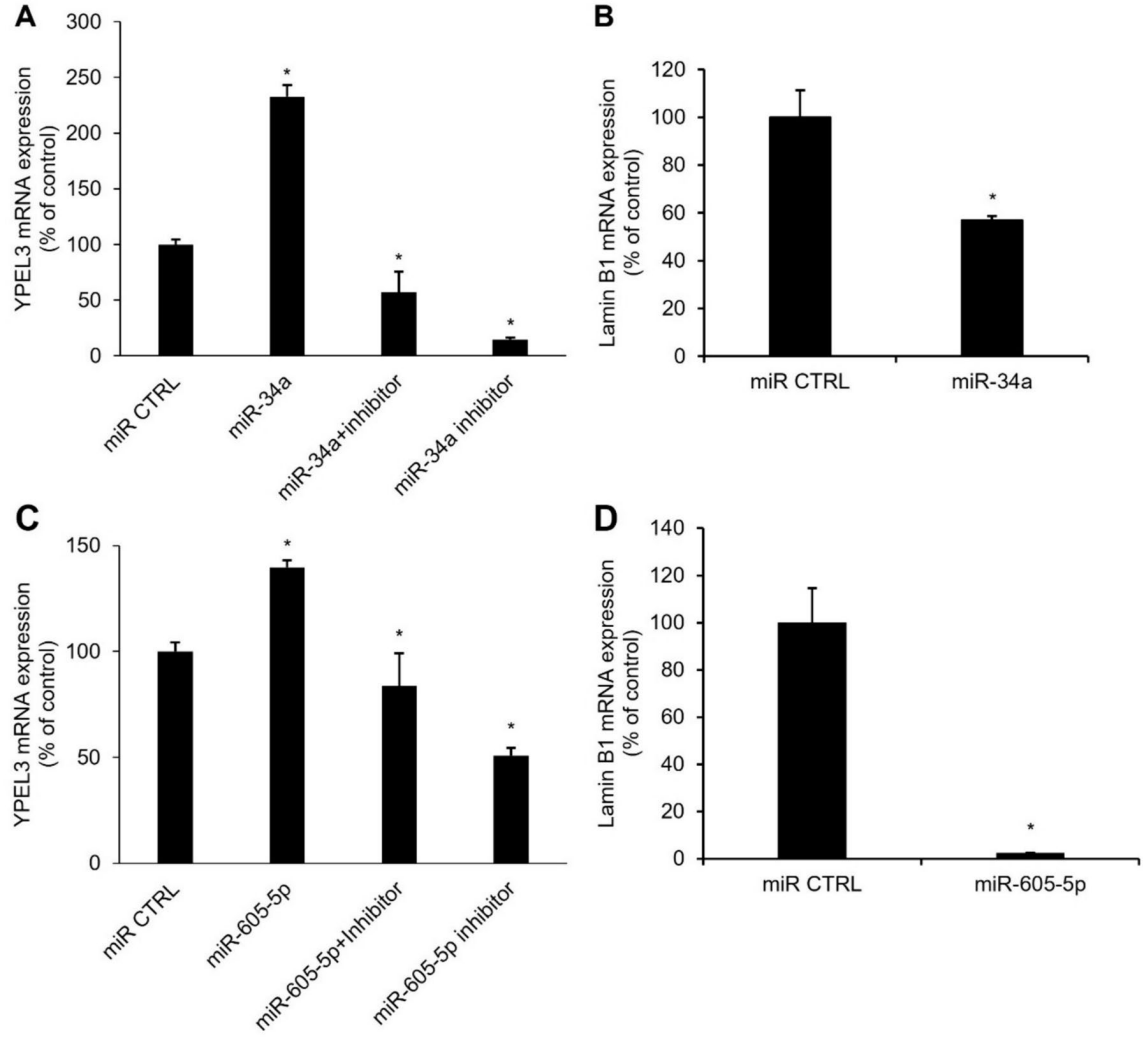
MiR-34a and miR-605-5p positively regulate YPEL3 expression in MCF-7 cells. **a** MiR-34a (80 nM) was transfected into MCF-7 cells in the presence or absence of miR-34a inhibitor (80 nM) and incubated for 48 h. Real-time qPCR was conducted to measure the mRNA expression of YPEL3. Data represent the mean ± SD (*n* = 3) (*p < 0.05). **b** MiR-34a was transfected into MCF-7 cells and incubated for 48 h. Real-time qPCR was conducted to measure the mRNA expression of lamin B1. Data represent the mean ± SD (*n* = 3) (*p < 0.05). **c** MiR-605-5p (80 nM) was transfected into MCF-7 cells in the presence or absence of miR-605-5p inhibitor (80 nM) and incubated for 48 h. Real-time qPCR was conducted to measure the mRNA expression of YPEL3. **d** MiR-605-5p was transfected into MCF-7 cells and incubated for 48 h. Real-time qPCR was conducted to measure the mRNA expression of lamin B1. Data represent the mean ± SD (*n* = 3) (*p < 0.05)

YPEL3 is a promising cellular senescence factor expressed via the action of p53 [22]. To elucidate whether the selected miRNAs can induce YPEL3 expression to promote cellular senescence, we treated the cells with miRNAs (miR-34a or miR-605-5p) for 48 h and performed qRT-PCR. YPEL3 mRNA expression significantly increased (~ 2.5 fold) in the cells transfected with miR-34a (80 nM) for 48 h. However, treatment with the mir-34a inhibitor strongly inhibited YPEL3 induction by miR-34a. Notably, treatment with the miR-34a inhibitor alone significantly suppressed YPEL3 mRNA expression (Fig. 4A). The expression of lamin B1 is downregulated by inducing cell senescence, and the loss of lamin B1 is a senescence-associated biomarker [23–25]. Treatment with miR-34a downregulated lamin B1 mRNA expression (Fig. 4B). YPEL3 mRNA expression were upregulated in the cells transfected with miR-605-5p (80 nM) for 48 h (~ 1.5 fold). Treatment with the mir-605-5p inhibitor strongly prevented YPEL3 induction by miR-605-5p. Moreover, treatment with the miR-605-5p inhibitor alone significantly suppressed YPEL3 mRNA expression (Fig. 4C). Lamin B1 mRNA expression was also suppressed by miR-605-5p (Fig. 4D).

### MiR-34a and miR-605-5p induce YPEL3 expression by suppressing YAP1 expression

**Fig. 5:**
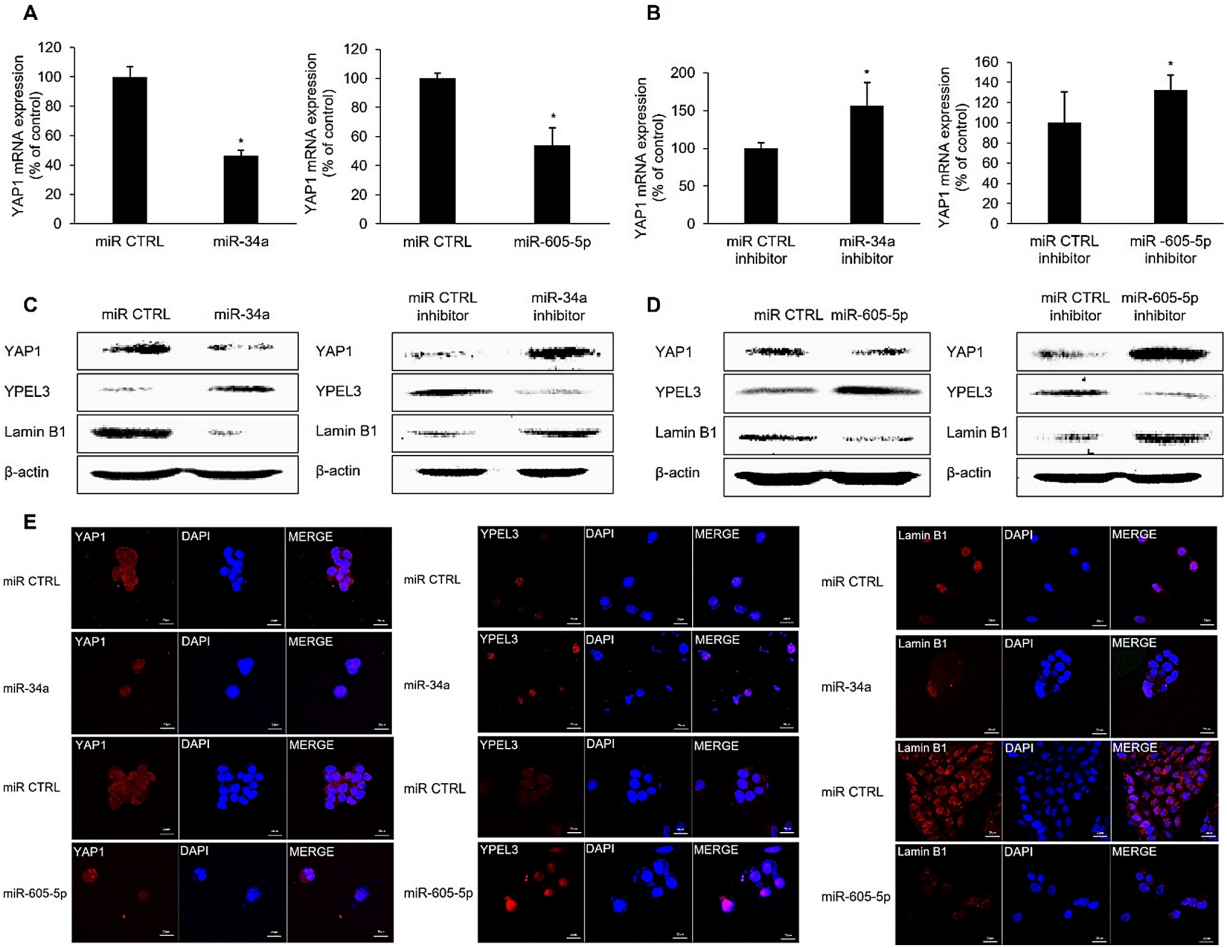
MiR-34a and miR-605-5p induce YPEL3 expression through YAP1 suppression. **a** Real-time qPCR was performed to measure the expression of YAP1 with a miR-34a or miR-34a inhibitor (80 nM). Data represent the mean ± SD (*n* = 3) (*p < 0.05). **b** Real-time qPCR was performed to measure the expression of YAP1 with miR-605-5p or miR-605-5p inhibitor (80 nM). Data represent the mean ± SD (*n* = 3) (*p < 0.05). **c** Cells were transfected with miR-34a or miR-34a inhibitor for 48 h. Total protein (40 µg) was subjected to western blot analysis with antibodies against YAP1, YPEL3, and lamin B1. β-actin was used as a loading control. **d** Cells were transfected with a miR-605-5p or miR-605-5p inhibitor for 48 h. Total protein (40 µg) was subjected western blot analysis with antibodies against YAP1, YPEL3, and lamin B1. β-actin was used as a loading control. Data represent the mean ± SD (*n* = 3) (*p < 0.05). **e** Confocal analysis was performed to detect YAP1, YPEL3, and lamin B1 expression after transfection with miR-34a and miR-605-5p. 4′,6-Diamidino-2-phenylindole was used for nuclear staining. Microscopy scale bar = 20 μm

Considering that YAP1 downregulation significantly enhances YPEL3 expression in MCF-7 cells [26], we determined the levels of YAP1 expression to identify the mechanisms by which these miRNAs upregulate YPEL3 expression. The YAP1/Hippo signaling pathway is a key regulator of organ size and tissue homeostasis, and its dysregulated expression is associated with cancer pathogenesis. YAP1 is a transcriptional coactivator of TEAD-mediated transcription and a well-established oncogene; its increased activity has been reported in human cancers [27]. Based on the miRNA database, YAP1 was identified as the target gene of miR-34a and miR-605. To investigate whether miRNAs regulate YAP1 expression, we transfected MCF-7 cells with miR-34a and miR-605-5p (80 nM). YAP1 mRNA expression was suppressed by miR-34a. Transfection with the miR-34a inhibitor induced YAP1 mRNA expression (Fig. 5A). Additionally, YAP1 mRNA expression was suppressed by miR-605-5p. YAP1 mRNA expression was promoted in the cells transfected with the miR-605-5p inhibitor (80 nM) for 48 h (Fig. 5B). Western blotting analysis showed that miR-34a induces YPEL3 protein expression. However, the protein levels of YAP1 and lamin B1 were decreased by miR-34a. The effect of the miR-34a inhibitor was determined to confirm the induction of YPEL3 expression by miR-34a. Transfection with the miR-34a inhibitor downregulated YPEL3 protein expression and upregulated YAP1 and lamin B1 protein expression (Fig. 5C). In addition, miR-605-5p enhanced YPEL3 protein expression and negatively regulated YAP1 and lamin B1 expression. Treatment with the miR-605-5p inhibitor exerted the opposite effect (Fig. 5D). Confocal micrographs showed that miR-34a and miR-605-5p downregulated YAP1 and lamin B1 protein expression and upregulated YPEL3 protein expression (Fig. 5E). These observations indicate that miRNAs, such as miR-34a and miR-605-5p, downregulate the expression of YAP1, which is a crucial cellular inhibitor of the Hippo pathway, leading to the upregulation of YPEL3 expression to promote cellular senescence and apoptosis. Moreover, miR-34a and miR-605-5p may function as tumor-suppressive miRNAs in MCF-7 cells by reducing YAP1 expression.

### MiR-34a and miR-605-5p induce cellular senescence and apoptosis by upregulating YPEL3 expression

**Fig. 6:**
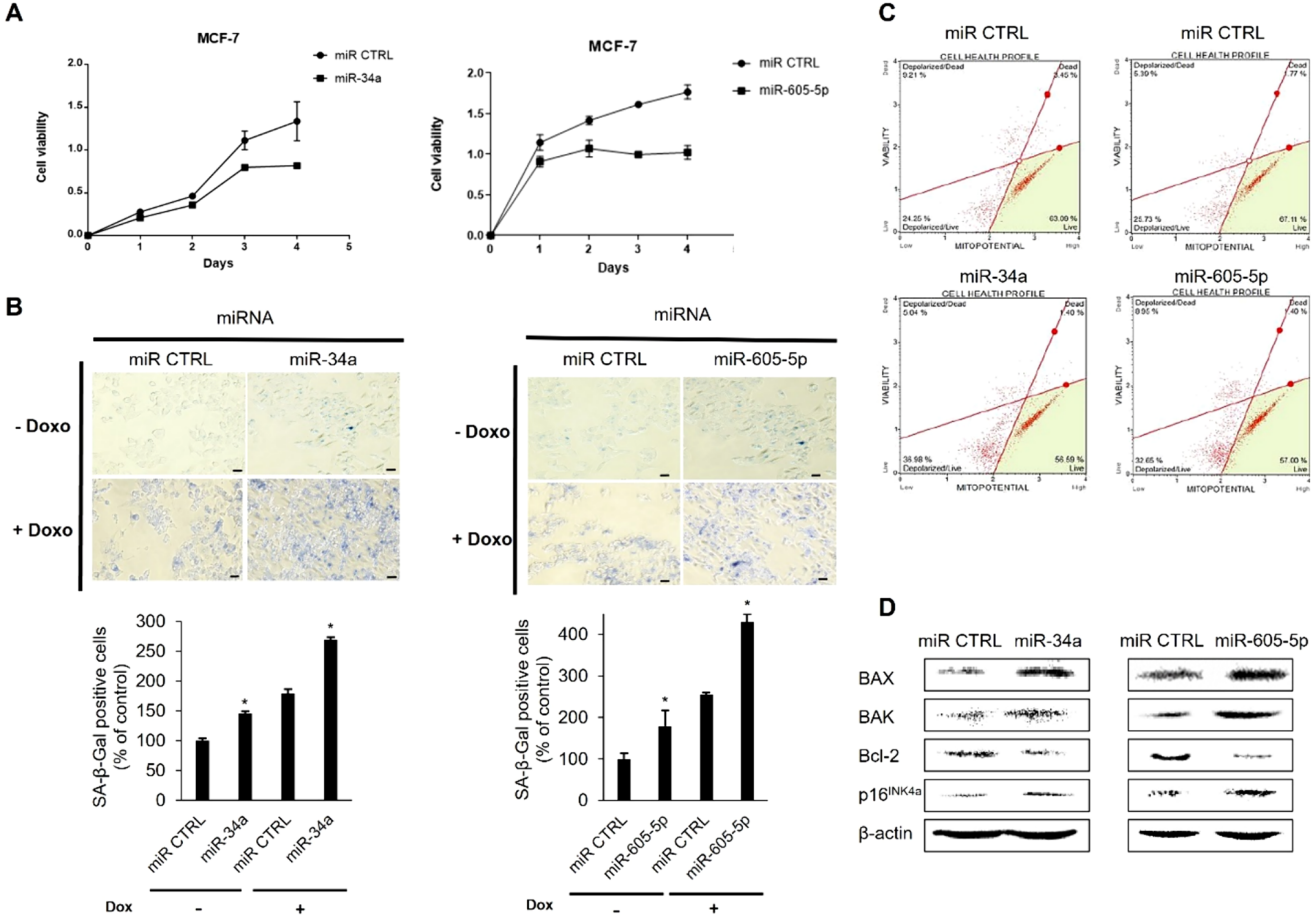
MiR-34a and miR-605-5p induce cell senescence and apoptosis in human breast cancer cells through YAP1 suppression and upregulation of YPEL3. **a** MCF-7 cells were transfected with miR-34a or miR-605-5p mimics (80 nM) and incubated for 4 days. Cell viability was measured at 450 nm using a microplate reader on the indicated days. Cell cultures were conducted in triplicate (*p < 0.05). **b** Senescence-associated β-galactosidase activity. Cellular senescence was measured by using a SA-β-gal kit after transfection with miR-34a or miR-605-5p (80 nM) in the presence or absence of doxorubicin (10 µM). Number of β-gal-positive cells was counted. Data represent the mean ± SD (*n* = 3) (*p < 0.05). **c** Cells were transfected with miR-34a or miR-605-5p mimics (80 nM) and incubated for 48 h. Cells were stained with 7-aminoactinomycin D. **d** Cells were prepared after transfection with miR-34a or miR-605-5p (80 nM) and then subjected to western blot with the relevant antibodies. β-actin was used as a loading control. Data represent the mean ± SD (*n* = 3) (*p < 0.05)

To confirm whether miR-34a and miR-605-5p act as tumor suppressors in MCF-7 cells, we examined their effects on cell proliferation. Cell viability was measured every 24 h for 4 days. Treatment with miR-34a or miR-605-5p decreased the rate of cell growth (Fig. 6A). Doxorubicin is currently considered one of the most potent therapeutic agents for breast cancer. It inhibits topoisomerase II and blocks DNA and RNA synthesis, resulting in apoptosis [28]. Doxorubicin-induced DNA damage eventually affects the function of p53, commonly known as a p53 activator, to enhance apoptosis [29]. Treatment with miR-34a slightly increased the number of β-galactosidase-positive cells. However, the number of β-galactosidase-positive cells significantly increased after treatment with miR-34a or miR-605-5p and doxorubicin. These results implied that miR-34a or miR-605-5p inhibited cell proliferation by inducing cellular senescence (Fig. 6B). MiR-34a and miR-605-5p significantly induce YPEL3 expression. Therefore, these data suggest that the induction of YPEL3 expression by miRNAs through the suppression of YAP1, MDM4, and MDM2 expression may inhibit cell proliferation. Cell senescence and early stages of apoptosis are strongly associated with mitochondrial dysfunction [30, 31]. Additionally, mitochondrial dysfunction caused by miRNAs is associated with cell senescence and the early stages of apoptosis. Transmembrane translocation, which is produced by the mitochondria, is related to the early stages of apoptosis, and depolarization of the internal mitochondrial membrane potential (∆Ψm) is an indicator of mitochondrial dysfunction [32]. In the present study, treatment with miR-34a or miR-605-5p increased the number of depolarized live cells by 10%–12% compared with the control cells. However, no significant difference in the number of depolarized dead cells was found (Fig. 6C). This result suggests that miR-34a and miR-605-5p promote the early stages of apoptosis or cellular senescence by altering the mitochondrial membrane potential. To determine whether these miRNAs cause mitochondrial apoptosis, we measured the levels of pro-apoptotic Bax, Bak, and p16^INK4a^ and anti-apoptotic Bcl-2. Treatment with miR-34a or miR-605-5p increased the levels of Bax and Bak but decreased the level of Bcl-2. However, it increased the level of p16^INK4a^, a cyclin-dependent kinase 4/6 inhibitor, indicating that these miRNAs may induce G1/S cell cycle arrest (Fig. 6D) [33, 34].

## Discussion

The roles of miRNAs in various cancer cells have become increasingly crucial and are still being studied. The notable effects of miR-34a on lung squamous cell carcinoma have been studied through suppressing cancer cell proliferation [35]. Previous studies have shown that miR-34a is involved in regulating cancer-related mechanisms and causes DNA damage by inhibiting the mRNA expression of target genes, thus acting as an essential mediator of tumor suppression [36, 37]. Many studies have also been conducted on miRNAs that antagonize various oncogenic processes, including the proliferation, migration, and invasion of tumor cells [38].

YPEL3 is a cellular aging factor that has not been studied sufficiently. Cellular senescence can be explained by the limited division capacity of normal cells, and it is increased by DNA damage through the loss of telomeres owing to continuous DNA replication. YPEL3 expression induced by DNA damage may be related to p53 level because p53-binding DNA response elements are located near the human YPEL3 promoter [22]. YPEL3 activates cellular senescence downstream of p53. Induction of YPEL3 reduces cell survival associated with increased cellular senescence by causing cell death. In the present study, we identified miRNAs that regulate YPEL3.

Several studies have demonstrated that YPEL3 is regulated by p53. Recently, a previous study newly showed that YPEL3 is inhibited by YAP1 [26]. Therefore, we investigated the relationship between miRNAs and YAP1. In addition, YAP1 is a target gene of miR-34a and is downregulated by miR-34a in vitro [39]. The Hippo pathway transduces multiple extracellular and intracellular signals and regulates cell proliferation, survival, apoptosis, and differentiation [40]. As a transcription factor-associated protein, YAP1 acts as a downstream effector in the Hippo pathway. Through this mechanism, YAP1 acts as an oncogene during cardiac development and regeneration [41]. Thus, YAP1 plays a key role in tumor formation, and several studies have been conducted to suppress its expression. In the present study, we confirmed that miR-34a and miR-605-5p act as tumor suppressors and upregulate YPEL3 expression by inhibiting YAP1 expression.

The mechanism by which YAP1 suppresses YPEL3 expression is fascinating because YAP/TAZ are known transcriptional coactivators that promote TEAD-mediated gene expression. Various target genes such as Cyr61, Myc, and CTGF are strongly activated by the nuclear translocation of YAP1. However, the genes inhibited by YAP1 are difficult to identify. A recent study has shown that nuclear TAZ binds to PPARγ and represses the expression of PPARγ and its target genes [42]. Determining the possible involvement of PPARγ in YPEL3 expression is necessary to understand the role of YAP1 in regulating YPEL3 gene expression.

**Fig. 7:**
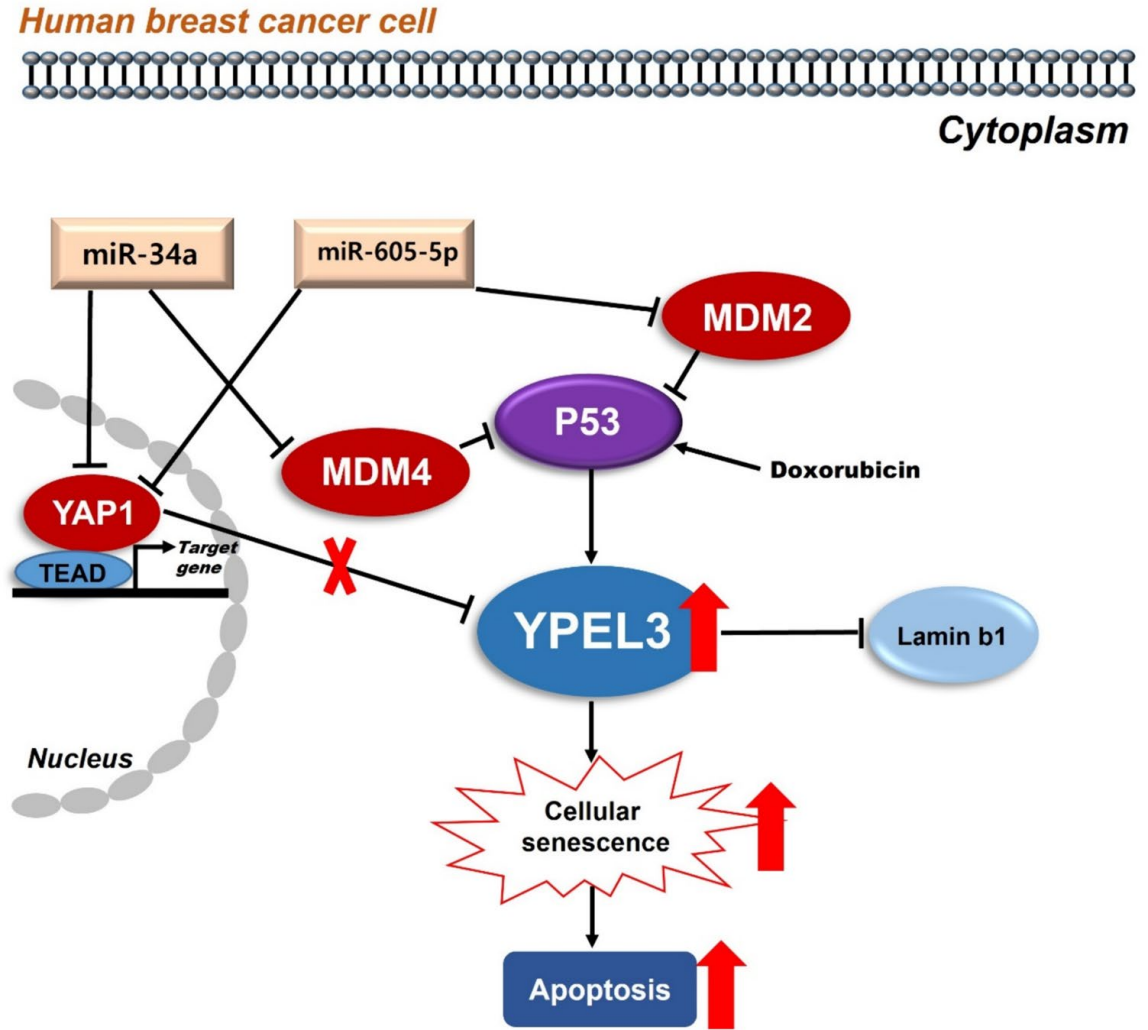
Schema depicting the relationship among microRNAs, YAP1, and YPEL3 that induce cellular senescence and apoptosis in MCF-7 cells

Collectively, the results of the present study indicate that the miRNAs-YAP1/YPEL3 cascade can be activated to protect cancer promotion. We report for the first time that miR-34a and miR-605-5p function as tumor suppressors by upregulating YPEL3 and downregulating YAP1 expression. Thus, miR-34a and miR-605-5p induce cellular senescence and apoptosis in human breast cancer cells by regulating the YAP1/Hippo pathway. Notably, we discovered novel miRNAs that target MDM4 and MDM2; this study enhances our understanding of the YAP1/Hippo pathway and the functions of miRNAs as novel therapeutic targets in breast cancer (Fig. 7). However, the molecular mechanisms underlying cellular senescence and apoptosis warrant further investigation.

## Supplementary Information

Below is the link to the electronic supplementary material.

Supplementary file1 (DOCX 400 kb)

## Data Availability

The Kaplan–Meier plot data supporting this study are available on the Kaplan–Meier Plotter website (https://kmplot.com/analysis/). Further information regarding this study can be provided by the corresponding author upon reasonable request.
