# Correction: Upregulation of YPEL3 expression and induction of human breast cancer cell death by microRNAs

**DOI:** 10.1007/s43188-024-00261-0

**Published:** 2024-08-29

**Authors:** Boyoung Lee, Yeo-Jung Kwon, Sangyun Shin, Tae-Uk Kwon, Hyemin Park, Hyein Lee, Ji-Heung Kwak, Young-Jin Chun

**Affiliations:** College of Pharmacy and Center for Metareceptome Research, Chung-Ang University, 84 Heukseok-Ro, Dongjak-Gu, Seoul, 06974 Republic of Korea

**Correction: Toxicol Res.** 10.1007/s43188-024-00251-2

In the funding information section of this article, one of the fundings NRF-2021R1A2C201239512 given for author Young-Jin Chun must be deleted and should have read Ministry of Science, ICT and Future Planning.

The original article has been corrected.

