# Supplementary material for: Upregulation of YPEL3 expression and induction of human breast cancer cell death by microRNAs
Source: Toxicol Res. 2024 Jun 21;40(4):599–611. doi: 10.1007/s43188-024-00251-2 (PMC11436705; doi:10.1007/s43188-024-00251-2)
Supplement: Supplementary file 1 — Supplementary file1 (DOCX 400 kb) [file 43188_2024_251_MOESM1_ESM.docx]

31th May 2024

Manuscript No.: TXRS-D-24-00057
Title: Upregulation of YPEL3 expression and induction of human breast cancer cell death by microRNAs
Article Type: Regular Paper
Journal Title: Toxicological Research
Corresponding Author: Professor Young-Jin Chun
All Authors: Boyoung Lee; Yeo-Jung Kwon; Sangyun Shin; Tae-Uk Kwon; Hyemin Park; Hyein Lee; Ji-Heung Kwak; Young-Jin Chun
Submit Date: Mar 10, 2024

Dear BBA - Molecular Basis of Disease Editor;

Thank you for your review letter of describing the review of our manuscript entitled “Upregulation of YPEL3 expression and induction of human breast cancer cell death by microRNAs” by Boyoung Lee, Yeo-Jung Kwon, Sangyun Shin, Tae-Uk Kwon, Hyemin Park, Hyein Lee, Ji-Heung Kwak, and myself. I am so grateful to have a chance to revise our manuscript. We are pleased to learn that the reviewers found our paper to be of interest and have revised the manuscript accordingly.

Response to the specific comments of the reviewers follow:

[Reviewer #1]

Reviewer #1: The present study investigated the involvement of microRNAs in YPEL3-mediated cell death in human breast cancer. Overall, this manuscript is well-structured, and its conclusions are substantiated by a sufficient amount of experimental evidence. However, there are some minor issues that need to be addressed prior to publication.

One notable concern pertains to the quality of the western blot images. The pixelated band patterns observed are not suitable for publication. Therefore, it is recommended to use high-resolution images to enhance clarity. Additionally, including protein size ladder indications would aid in facilitating clear demonstration.
 **A)** All western blot images have been quality improved through increased resolution.

To further validate the miRNA-mediated mechanisms elucidated in this study, mutation studies are warranted to confirm the miRNA binding sequences on the target genes (within the 3'-UTR of mRNA). These experimental data could be incorporated into either Figure 3 or 4. Alternatively, discussing the limitations of the study and suggesting avenues for further research would also be beneficial.

**A)**

The inhibitors that we used targeting miR-34a and miR-605-5p in this article are synthesized with the antisense sequence to the mature miR-34a and miR-605-5p, respectively. Since the purpose of mutational studies on miRNAs is to demonstrate whether the miRNA acts through its binding ability to the target gene, our studies using inhibitors that interfere with the action of miRNAs through full antisense binding to them are useful as alternative tools for mutation studies.

The authors need to receive professional English language service to correct grammar and other errors in the manuscript and the Certificate of editing must be enclosed in the supplementary file, if it was not provided in the first submission. The authors should provide DOIs for the articles they cite. (submission guidelines).

This manuscript received the professional English language service and got the certificate for that. The certificate for editing has been attached here. DOIs for references have been added accordingly.

Reviewer #2: The authors seem to have a firm grasp on the molecular mechanisms involving tumor suppressing miRNAs and cancer.
MicroRNA-34 mimics are on a phase I clinical trial for various types of cancer and recently, research into miRNAs as targeted therapeutics seem most on topic.
The paper is well-constructed and reads smoothly.
I have just one suggestion that is quite minor - Merging Figures 3 and 4, as is the data shown in Figure 5, would result in a more compact figure list.

A) Figure 3 and 4 have been merged and the manuscript has been revised accordingly.

We feel that we have responded to the points raised by the reviewers and the revised manuscript should prove to be suitable for publication. However, if you feel that further changes are in order, please do not hesitate to contact me. Thank you and the reviewers for all your efforts and best regards.

Corresponding Author,

Young-Jin Chun, Ph. D.
